# Promoting the Avoidance of High-Calorie Snacks: Priming Autonomy Moderates Message Framing Effects

**DOI:** 10.1371/journal.pone.0103892

**Published:** 2014-07-31

**Authors:** Louisa Pavey, Sue Churchill

**Affiliations:** 1 Kingston University, Kingston-upon-Thames, United Kingdom; 2 University of Chichester, Chichester, United Kingdom; University of Bath, United Kingdom

## Abstract

The beneficial effects of gain-framed vs. loss-framed messages promoting health protective behaviors have been found to be inconsistent, and consideration of potential moderating variables is essential if framed health promotion messages are to be effective. This research aimed to determine the influence of highlighting autonomy (choice and freedom) and heteronomy (coercion) on the avoidance of high-calorie snacks following reading gain-framed or loss-framed health messages. In Study 1 (*N* = 152) participants completed an autonomy, neutral, or heteronomy priming task, and read a gain-framed or loss-framed health message. In Study 2 (*N* = 242) participants read a gain-framed or loss-framed health message with embedded autonomy or heteronomy primes. In both studies, snacking intentions and behavior were recorded after seven days. In both studies, when autonomy was highlighted, the gain-framed message (compared to the loss-framed message) resulted in stronger intentions to avoid high-calorie snacks, and lower self-reported snack consumption after seven days. Study 2 demonstrated this effect occurred only for participants to whom the information was most relevant (BMI>25). The results suggest that messages promoting healthy dietary behavior may be more persuasive if the autonomy-supportive vs. coercive nature of the health information is matched to the message frame. Further research is needed to examine potential mediating processes.

## Introduction

Message framing is a health promotion strategy which communicates health benefits (a gain-framed message) or health costs (a loss-framed message), depending on the features of the target health behavior [1] [2]. Prevention or protection behaviors such as sunscreen use, exercise, and diet (e.g., fruit and vegetable consumption and reduced fat intake) are unlikely to lead to negative health outcomes and consequently pose low risk to individuals. In contrast, detection behaviors such as health screening (e.g., mammography and HIV testing) are considered risky as they could lead to diagnosis of serious illness. According to Prospect Theory, people are risk-seeking when considering potential gains, and risk-averse when considering potential losses [3] [4]. There is some support for the notion that gain-framed messages are more effective than loss-framed messages when encouraging ‘lower-risk’ prevention or protection behaviors, whereas loss-framed messages are more effective than gain-framed messages for ‘higher-risk’ illness detection behaviors [1] [5] [2]. Thus, people tend to be more persuaded by information about the benefits of eating a healthy diet vs. the negative consequences of not eating a healthy diet (a lower-risk, prevention behavior), whereas people tend to be more persuaded when given information about the potential negative consequences of not attending a health screening vs. the benefits gained from attending a screening (a higher-risk, detection behavior). However, mixed findings in the literature suggest that Prospect Theory may be limited in its ability to fully explain the differential effects of gain-framed or loss-framed messages, particularly in health promotion settings.

For example, a recent meta-analysis [6] confirmed that compared to loss-framed messages, gain-framed messages conferred a weak but significant advantage for encouraging prevention behaviors such as sun-screen use, smoking cessation, physical activity, and diet, with no overall effect of message frame found for detection behaviors. However, studies have found no beneficial effect of gain-framed vs. loss-framed messages for prevention behaviors [7] [8] [9] [10] and other meta-analyses demonstrate inconsistent results in the literature [11] [12]. This poses difficulties for both researchers and practitioners, as it is not clear whether the effectiveness of highlighting potential gains vs. losses is dependent on the type of health behavior targeted. Authors have proposed that these mixed findings warrant investigation of the specific conditions in which gain-framed or loss-framed messages are most effective [13].

In order to enhance our understanding of whether gain-framed or loss-framed messages are effective in eliciting a desirable health behavior, it is therefore necessary to consider potential moderating conditions. Research has shown both individual and contextual moderators to influence the extent to which gain-framed or loss-framed messages are more effective [14] [15]. For example, positive and negative mood states have been found to moderate the effectiveness of gain-framed and loss-framed messages, with gain-framed messages (compared to loss-framed messages) eliciting greater health-recommended behavior when the recipient was in a positive compared to negative mood [16]. The emotional states of fear and anger have also been found to influence the success of gain-framed and loss-framed messages promoting fruit and vegetable consumption: gain-framed messages (compared to loss-framed messages) were more effective when the recipient was angry compared to afraid [17]. The persuasiveness of gain-framed and loss-framed messages has also been found to depend on the self-efficacy of the person reading the message [18], and their approach vs. avoidance motivational orientation [19].

Previous research has suggested that identifying individual difference moderators may be useful to help develop individually tailored messages to encourage health behaviors [15]. Although this is a desirable objective, it may not always be feasible to individually tailor messages to suit individuals on the basis of their emotional state, motivational orientation, or self-efficacy. In addition, this strategy may be costly and time-consuming for practitioners. A more efficient solution may be to examine whether an individual difference that might facilitate message acceptance could be boosted to increase message effectiveness, and for this manipulation to be incorporated within the framed message to increase persuasion.

One moderator of message framing effects which may be particularly suited to this approach concerns individual differences in feelings of autonomy. Self-determination Theory suggests that autonomy, defined as the experience of engaging in volitional action based on personal interests and values, is proposed to be one of our basic psychological needs (in addition to competence and relatedness) that when frustrated can lead to maladaptive psychological functioning and lower well-being [20]. Research has shown that higher levels of autonomy are associated with greater autonomous motivation and intentions to reduce unhealthy behavior following reading health-risk information [21] [22]. In addition, a large body of literature suggests that increased feelings of autonomy are associated with greater autonomous motivation and greater adherence to recommended health behaviors including diet [23] [24], exercise [24] [25], smoking cessation [26], and diabetes care [27]. Autonomy-supportive styles of persuasion have also been found to be more effective in encouraging people to accept health advice about quitting smoking than more dictatorial styles [28].

With particular relevance to the current research, a recent study demonstrated that levels of autonomy were only associated with stronger intentions to consume fruit and vegetables, and greater fruit and vegetable consumption seven days later, when the recipient was given gain-framed (vs. loss-framed) health information [29]. This may be due to an autonomous individual construing the behavior as in accordance with their interests and values. As such, greater autonomous motivation to conduct a health behavior may result in perceptions of the behavior as less risky (i.e., there is no perceived risk of attempting but failing to comply as conducting the behavior will benefit the individual both in terms of their health outcome and in the inherent satisfaction gained from acting autonomously). It may also serve to reduce the extent to which the information is viewed as risky to their self-integrity (i.e., if the behavior is conducted autonomously then complying with the message would not compromise autonomy or self-integrity). Thus, autonomy is likely to increase positive affective and behavioral responses to gain-framed messages and lead to greater motivation to adhere to the recommended health behavior. This proposition is supported by research which showed a reduction in the classic loss aversion effect under conditions of high autonomy [30]. In this experiment, contextual autonomy support (vs. no autonomy support) led to greater persistence on a word task when the gains (vs. losses) of the participant’s previous performance were communicated.

Research suggests that feelings of autonomy can be experimentally manipulated using non-conscious priming methods. Using sentence unscrambling tasks, previous researchers [31] have developed a method for priming both autonomy and the opposite of autonomy: heteronomy (i.e., pressure and coercion from other people). Experimentally highlighting autonomy using this priming method has been found to reduce defensiveness [32] and increase the effectiveness of health-risk information encouraging moderate alcohol consumption, particularly for those most at risk [33]. This has been suggested to be due to autonomy reducing the motivation to respond with defensiveness (e.g., denial, avoidance, or justification) to information which has the potential to threaten self-worth and self-integrity [33] [34].

Consistent with Prospect Theory, we predict that increased motivation to engage in a low-risk preventative behavior such as avoiding snacking would occur when the potential gains (vs. losses) of that behavior are communicated [1] [2] [5]. In addition, following previous research showing that individual differences in autonomy moderate message framing effects for the low-risk preventative health behavior of fruit and vegetable consumption [29], and research suggesting that autonomy supportive context increase response to gain-framed communications [30], we predict that the gain-framed information may be particularly effective under conditions of high autonomy (vs. neutral conditions or those which highlight heteronomy).

The current research investigated whether highlighting feelings of autonomy increased the persuasiveness of information framed in terms of the potential health gains of avoiding an unhealthy behavior vs. the potential health losses associated with not avoiding an unhealthy behavior. This presents a novel use of experimental manipulation to demonstrate the influence of autonomy on responses to gain-framed vs. loss-framed messages. In addition to the theoretical implications of the research, the results have substantial practical implication for the design of health messages, and could inform the decision to use an autonomy-supportive or more dictatorial style of presenting health advice to enhance persuasion following reading gain-framed vs. loss-framed health messages.

## Study 1

In Study 1 the moderating effect of an autonomy and heteronomy priming task on participants’ intentions to avoid high-calorie snacks and their subsequent snacking behavior was tested. For autonomy-primed individuals, it was predicted that a gain-framed message would result in stronger intentions to avoid high-calorie snacks and less subsequent snacking behavior compared to a loss-framed message.

### Method

#### Participants and design

A 3×2 (Prime [autonomy, neutral, heteronomy]×Frame [gain, loss]) experimental design was used with participants randomly allocated to one of the six conditions. Participants were 152 university students (120 females and 32 males) aged 20 to 61 (*M* = 27.39, *SD* = 6.59) who participated as part of their class requirements (participants were not compensated for their time). Of the 152 participants recruited at Time 1, 52 females and 10 males completed the questionnaire at Time 2 (with the drop-out rate due to the scheduling of the seminar classes and non-attendance at seminars in the second session). There were no significant differences in age, gender, baseline snacking behavior, baseline intentions, baseline autonomy, or condition, between those who completed only the Time 1 questionnaire and those who completed both parts of the study (all *p*’s>.10).

#### Materials and procedure

Participants were invited to complete the initial online questionnaire in groups of 15–20 people during seminar classes. Baseline snacking behavior was measured with a single-item [15]: “How many times did you eat high-calorie snacks over the last 7 days?” with response options of: 1 = *Not at all*; 2 = *Once a week*; 3 = *2–4 times a week*; 4 = *5–6 times a week*; 5 = *Once per day*; 6 = *2–3 times each day*; 7 = *4 or more times each day*. Individual differences in autonomy at baseline were measured using the Basic Needs Satisfaction Scale (Autonomy Subscale: 9 items, e.g., “I feel that my choices are based on my true interests and values,” measured on a 1–7 scale from *not at all true for me* to *very true for me*, α = .82). Baseline intentions to avoid high-calorie snacks were measured with the single item: “I intend to avoid eating high-calorie snacks over the next seven days,” with response options on a 1–7 scale from *not at all true* to *very true*.

Participants then completed an autonomy, neutral, or heteronomy sentence unscrambling task [31]. In all conditions, participants were given a list of 30 sets of five words, and for each set were asked to make a sentence out of four of the five words. In the autonomy prime condition, 15 out of the 30 sentences contained autonomy-related words (e.g., freedom, choice, and decision), and in the heteronomy prime condition, 15 out of the 30 sentences contained heteronomy related words (e.g., pressure, control, and must). In the neutral prime condition all words were unrelated to autonomy and heteronomy (e.g., book, table, and coffee). As a manipulation check, participants were then given eight partially completed words and were asked to complete them with the first word that came to mind. Five of the words could be completed to form a word related to autonomy (e.g., “sel _ _ _” could be completed as “select”), and participants were given a score of 0–5 indicating how many autonomy-related words were completed. Following the prime, participants read a health message (see Figure S1) which was identical except for details of either the benefits of avoiding snacking (gain-framed), or the health dangers of not avoiding snacking (loss-framed). The health messages were constructed following research that has used similar messages to successfully differentiate the effects of gain-frames and loss-frames for a range of health behaviors [1] [29].

Participants’ Time 1 intentions to avoid eating unhealthy snacks over the next 7 days were measured with three items completed directly after the health message (e.g., “I intend to avoid eating high calorie snacks over the next 7 days”, with responses on a 1–7 scale from *strongly disagree* to *strongly agree*), α = .90. Seven days later, participants completed a second questionnaire. Time 2 intentions to avoid eating high-calorie snacks were measured using the same three items as at Time 1, α = .86. Time 2 snacking behavior was measured using the same item as at baseline. Participants were then thanked and debriefed.

#### Ethics Statement

Ethical guidelines were followed in the conduct of this research. The first page of the online questionnaire gave full details of the study and informed participants about their right to withdraw from the research at any time. Participants were not able to access the questionnaire until they had ticked a box giving their consent to participate. This consent was recorded with the other research data. The research and consent procedure was approved by the Kingston University Faculty Ethics Committee prior to data collection.

### Results

A One-way ANOVA indicated the expected effect of Prime (autonomy, neutral, heteronomy) on the manipulation check task, *F*(2, 113) = 8.49, *p*<.001, ηρ^2^ = .13. Autonomy-primed participants completed a greater number of words related to autonomy (*M* = 2.86, *SE* = 0.19) than neutral- (*M* = 2.46, *SE* = .18) and heteronomy-primed participants (*M* = 1.83, *SE* = .17), linear contrast, *F*(1, 113) = 16.41, *p*<.001. Three 3 (Prime [autonomy, neutral, heteronomy]×2 (Frame [gain, loss]) ANCOVAs were then conducted to examine the effects of Prime and Frame on intentions to avoid high calorie snacks at Time 1 and Time 2, and snacking behavior at Time 2, with baseline intentions, baseline snacking behavior, and baseline autonomy added as covariates. Estimated marginal means are shown in Table 1.

**Table 1:** **Study 1: Estimated marginal means and standard errors of each dependent variable for autonomy, neutral, and heteronomy prime participants who read a gain-framed and loss-framed health message.**

	Gain-frame						Loss-frame					
	Autonomy		Neutral		Heteronomy		Autonomy		Neutral		Heteronomy	
	*M*	*SE*	*M*	*SE*	*M*	*SE*	*M*	*SE*	*M*	*SE*	*M*	*SE*
Time 1 Intentions to avoid snacking	4.26	.25	3.81	.24	3.50	.23	3.33	.24	3.74	.23	3.88	.23
Time 2 Intentions to avoid snacking	4.24	.35	3.53	.32	3.39	.36	2.80	.34	3.47	.35	4.00	.37
Snacking behavior*	3.29	.32	4.08	.29	3.80	.32	4.16	.30	3.73	.32	2.83	.33

*Lower scores for behavior indicate greater avoidance of high-calorie snacks.

#### Predicting intentions

For Time 1 intentions, there was no main effect of Prime, *F*(2, 141) = 0.11, *p* = .894, ηρ^2^<.01, or Frame *F*(1, 141) = 1.07, *p* = .302, ηρ^2^<.01. However, there was a significant Prime×Frame interaction, *F*(2, 143) = 4.01, *p* = .022, ηρ^2^ = .05. Simple main effects analysis indicated that among autonomy-primed participants, gain-framed message participants reported higher Time 1 intentions than loss-framed message participants, *F*(1, 141) = 7.09, *p* = .009, ηρ^2^ = .05. Among neutral-primed and heteronomy-primed participants, there was no difference between the gain-framed and loss-framed messages, *p’s*>.10.

For Time 2 intentions, there was also no main effect of Prime, *F*(2, 60) = 0.17, *p* = .843, ηρ^2^<.01, or Frame, *F*(1, 60) = 1.08, *p* = .303, ηρ^2^ = .02, but there was a significant Prime×Frame interaction, *F*(2, 60) = 4.47, *p* = .015, ηρ^2^ = .13. Simple main effects analysis indicated that among the autonomy-primed participants, gain-framed message participants reported higher Time 2 intentions than loss-framed message participants, *F*(1, 60) = 8.59 *p* = .005, ηρ^2^ = .13. For neutral-primed and heteronomy-primed participants, there was no difference between the gain-framed and loss-framed messages, *p’s*>.10.

#### Predicting behavior

**Figure 1:**
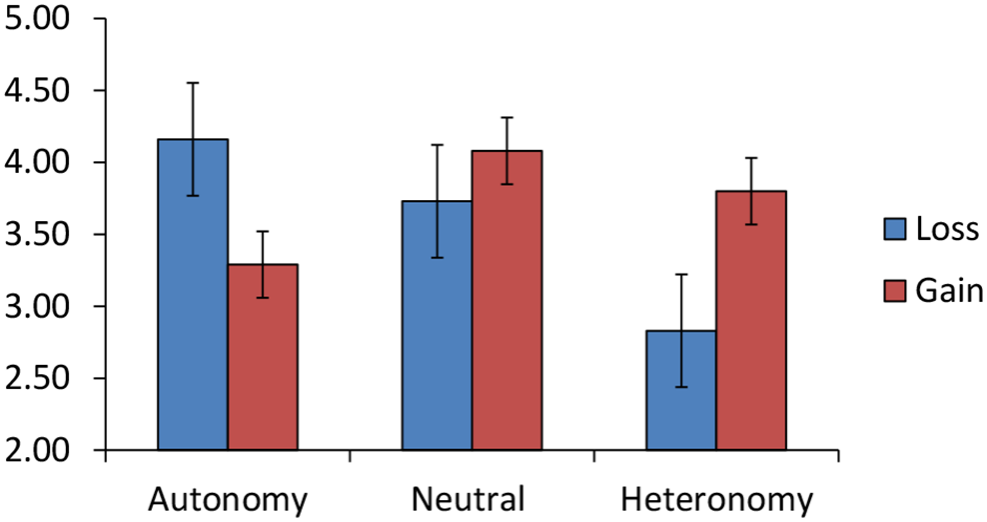
Study 1: Prime×Frame interaction for snack food consumption. Estimated marginal means (+−*SE*) of Time 2 snack food consumption for autonomy, neutral or heteronomy prime participants who read a gain-framed or loss-framed health message.

For Time 2 snacking behavior, there was no main effect of Prime, *F*(2, 60) = 1.71, *p* = .190, ηρ^2^ = .05, or Frame, *F*(1, 60) = 0.35, *p* = .556, ηρ^2^<.01, but there was a significant Prime×Frame interaction, *F*(2, 60) = 4.48, *p* = .015, ηρ^2^ = .13. Simple main effects analysis indicated that of the autonomy-primed participants, gain-framed message participants reported marginally lower snack consumption at Time 2 compared to loss-framed message participants, *F*(1, 60) = 3.86, *p* = .054, ηρ^2^ = .06. For neutral-primed participants, there was no difference between the gain-framed and loss-framed messages, *p*>.10. Of the heteronomy-primed participants, gain-framed message participants reported greater snack consumption at Time 2 compared to loss-framed message participants, *F*(1, 60) = 4.63, *p* = .035, ηρ^2^ = .07. Estimated marginal means for this interaction are displayed in Figure 1.

### Discussion

The results of Study 1 support our hypotheses, and suggest that the persuasiveness of gain-framed and loss-framed health messages depends on whether autonomy is highlighted. No main effect of Frame condition was found, supporting previous research which has indicated inconsistent support for the application of Prospect Theory in health promotion settings. In addition, no main effect of Prime was found, suggesting that although research has shown that priming autonomy increases the effectiveness of health-risk information, [33], this may only be true when information about the potential gains of a health behavior is conveyed. For participants primed with autonomy, the gain-framed message was more effective in promoting the avoidance of high-calorie snacks, compared to the loss-framed message. This finding indicates that to increase the effectiveness of health promotion information about the avoidance of snacking, the information presented should be both gain-framed and autonomy supportive.

Unexpectedly however, we also found that for participants primed with heteronomy, the loss-framed message was more effective in reducing the consumption of high-calorie snacks than the gain-framed message, although no significant effects were found for intentions. This effect of frame condition for heteronomy primed participants may have been due to the heteronomy prime increasing participants’ perception of the behavior as risky, thus rendering the loss-framed message more effective. Further research is needed to corroborate this finding and explore why the effect might only have occurred for behavior and not for intentions (for example, by eliciting a more implicit, direct effect on behavior). In addition, the lowest intentions to avoid snacking, and greatest snacking behavior, were found for those participants who were primed with autonomy and read the loss-framed message. It is possible that the disparity between the autonomy of the individual and the loss-frame (which could be viewed as more coercive), may have resulted in a reactance or boomerang effect, with participants engaging in freedom-restoring responses causing them to reject the message [35]. Thus, the congruency between the prime and frame conditions may be driving this effect. The process driving the autonomy and framing interaction may therefore be similar to the congruency effect reported for participant’s approach vs. avoidance motivational orientation and message frame [19]. Further research is needed to clarify the process underlying the interaction between the prime and frame conditions.

Although the results of Study 1 supported our hypotheses, there are a number of limitations to consider. For example, a relatively small sample of participants was used, and the majority of participants were female. Previous research has shown that females may be more sensitive to message framing effects, particularly for gain-framed messages in a health promotion context [36] [37]. Therefore, the effects we find for females in Study 1 may not be present for men. In addition, Study 1 did not measure weight or height, therefore we could not determine whether these effects would be particularly prominent for those to whom the information is most relevant (i.e., for participants who are overweight or obese). The measure of snacking behavior could also be improved, as the measure used in Study 1 assumed participants ate a similar number of snacks each day. The likert scale used to measure snacking behavior in Study 1 was therefore replaced with a continuous frequency measure in Study 2. Furthermore, the priming task used in Study 2 may not lend itself to being used in practice: asking people to complete a priming task prior to reading a health message is likely to be unfeasible. Study 2 aimed to address these limitations and increase the extent to which these findings could be applied within a healthcare context.

## Study 2

To strengthen the conclusions of Study 1, and increase the practical applicability of the findings, Study 2 sought to replicate the results of Study 1 and to determine whether autonomy and heteronomy primes that were embedded within the gain-framed and loss-framed messages could influence participants’ snacking intentions and behavior. Thus, instead of activating feelings of autonomy and heteronomy using a sentence unscrambling task, in Study 2 the wording of the gain-framed and loss-framed health messages was changed to convey either choice (autonomy) or coercion (heteronomy). It was hypothesized that those who read a gain-framed message with embedded autonomy-primes would report stronger intentions to avoid snacking, and reduced snacking seven days later, compared to those who read a loss-framed message with embedded autonomy-primes. Based on the findings of Study 1, it was also possible that those who read a gain-framed message with embedded heteronomy-primes would report lower avoidance of snacking, compared to those who read a loss-framed message with embedded heteronomy-primes. In addition, the limitations of Study 1 were addressed by using a larger, non-student sample with a wider age range and more equal balance in gender.

In accordance with previous research [33], the effect of highlighting autonomy may be particularly pronounced when the person perceives the information as personally relevant and self-threatening. Research examining the moderating impact of personal relevance on message framing effects has shown that people may engage in more systematic processing when a message is of high personal relevance, and that message framing effects are particularly pronounced under these conditions [38] [14]. It has been suggested that the advantage of gain-framed messages for promotion behaviors, and loss-framed messages for prevention behaviors, may be amplified when the message is of high personal relevance [1] [14]. Therefore Study 2 also sought to determine whether the interaction between prime and message frame was present particularly for those to whom the information was most relevant (i.e., for participants who were overweight or obese).

### Method

#### Design and participants

A 2 (Embedded prime [autonomy, heteronomy])×2 (Frame [gain, loss])×2 (BMI [normal vs. overweight]) design was used with participants randomly allocated to one of the four experimental conditions. Participants were allocated to the normal weight or overweight groups based on World Health Organization guidelines. Those allocated to the normal weight category indicated a BMI (Body Mass Index = kg/m^2^) within the healthy range of less than 25 (*n* = 91), and those allocated to the overweight category indicated a BMI in the overweight or obese category of 25 or higher (*n* = 148, four missing values for BMI).

Participants (*N* = 243) were 112 females and 128 males (three missing values for gender), recruited via an online survey hosting company who reward survey participants with points which can be exchanged for consumer vouchers. Participants were aged 20 to 70 (*M* = 27.39, *SD* = 6.59), with a BMI between 17.30 and 53.13 (*M* = 27.01, *SD* = 6.58). Of the 243 participants recruited at Time 1, 196 completed the questionnaire at Time 2. There were no significant differences in age, gender, BMI, baseline snacking behavior, baseline intentions, baseline autonomy, or condition, between those who completed only the Time 1 questionnaire and those who completed both parts of the study.

#### Materials and procedure

Participants completed the questionnaires online by clicking on a link sent via email. The baseline snacking behavior item used in Study 1 was modified in Study 2 to provide a more sensitive measure. Baseline snacking was measured with the open response frequency item: “How many high-calorie snacks have you eaten in the past 7 days? This can include chocolate, crisps, cake, pastries, biscuits, and other unhealthy sweet or savory snacks.” Individual differences in autonomy at baseline were measured using the same items as in Study 1, α = .80. Baseline intention to avoid high-calorie snacks was measured with the same item as in Study 1. Participants were then asked to read the same health messages as in Study 1 with words related to autonomy or heteronomy embedded within them (see Figure S2).

Participants’ Time 1 intentions to avoid eating unhealthy snacks over the next 7 days were measured with the same three items as in Study 1, completed directly after the health message, α = .98, and participants were also asked to report their weight and height, and to give their email address. Seven days later, participants were emailed the link to the second questionnaire. Time 2 intentions to avoid eating high-calorie snacks were measured using the same three items as at Time 1, α = .86. Time 2 snacking behavior was measured using the same item as at baseline. Participants were then thanked and debriefed.

#### Ethics Statement

Ethical guidelines were followed in the conduct of this research. The first page of the online questionnaire gave full details of the study and informed participants about their right to withdraw from the research at any time. Participants were not able to access the questionnaire until they had ticked a box giving their consent to participate. This consent was recorded with the other research data. The research and consent procedure was approved by the Kingston University Faculty Ethics Committee prior to data collection.

### Results

Three 2 (Embedded Prime [autonomy, heteronomy]×2 (Frame [gain, loss])×2 (BMI [normal, overweight]) ANCOVAs were conducted on intentions to avoid high calorie snacks at Time 1 and Time 2, and snacking behavior at Time 2, with baseline intentions, baseline snacking behavior, baseline autonomy, BMI, and gender added as covariates. Estimated marginal means for each variable in each condition are shown in Table 2.

**Table 2:** **Study 2: Estimated marginal means and standard errors of each dependent variable for high BMI embedded autonomy-prime and embedded heteronomy-prime participants who read a gain-framed and loss-framed health message.**

	Gain-frame				Loss-Frame			
	Autonomy		Heteronomy		Autonomy		Heteronomy	
	*M*	*SE*	*M*	*SE*	*M*	*SE*	*M*	*SE*
Time 1 Intentions to avoid snacking	4.91	.19	4.32	.18	4.36	.19	4.85	.18
Time 2 Intentions to avoid snacking	4.99	.32	4.41	.27	3.93	.28	4.87	.27
Snacking behavior*	4.86	1.02	7.36	.87	5.92	.90	4.74	.85

*Lower scores for behavior indicate greater avoidance of high-calorie snacks.

#### Predicting intentions

**Figure 2:**
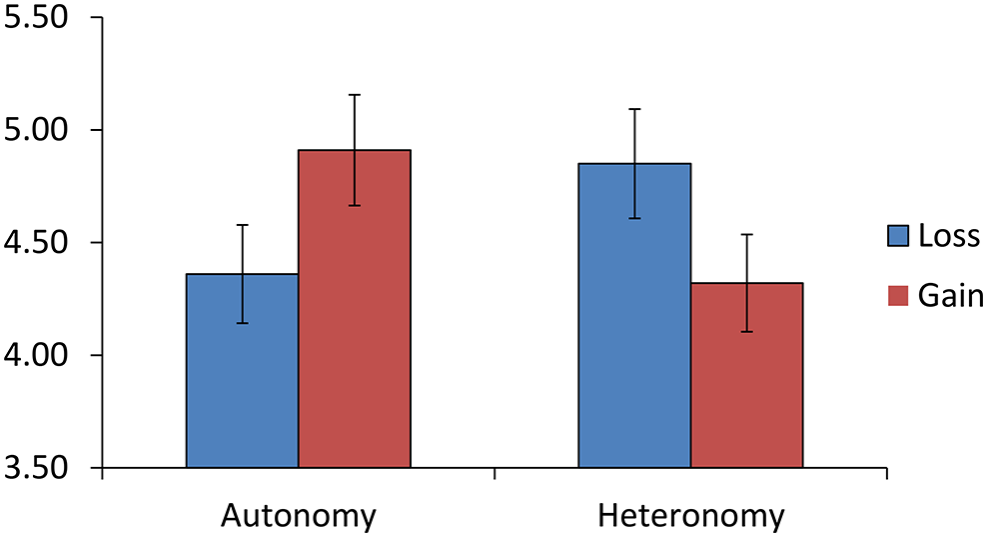
Study 2: Prime ×**Frame interaction for intentions to avoid high calorie snacks.** Study 2: Estimated marginal means (+−*SE*) of Time 1 intentions to avoid high calorie snacks in the next 7 days for embedded autonomy-prime or embedded heteronomy-prime participants who read a gain-framed or loss-framed health message.

For Time 1 intentions, there was no main effect of Embedded Prime, *F*(1, 223) = 0.78, *p* = .380, ηρ^2^<.01, or Frame *F*(1, 223) = 0.55, *p* = .461, ηρ^2^<.01, and no significant Embedded Prime×Frame interaction, *F*(1, 223) = 2.48, *p* = .117, ηρ^2^ = .01. However, there was a significant Embedded Prime×Frame×BMI interaction, *F*(1, 223) = 4.47, *p* = .036, ηρ^2^ = .02. For participants who were overweight, there was a significant Embedded Prime×Frame interaction, *F*(1, 142) = 7.30, *p* = .008, ηρ^2^ = .05, whereas there was no significant Embedded Prime×Frame interaction for participants who were normal weight, *F*(1, 85) = 0.91, *p* = .764, ηρ^2^<.01. Simple main effects analysis indicated that among participants who were overweight and who read the embedded autonomy-prime message, gain-framed message participants reported stronger intentions than loss-framed message participants, *F*(1, 223) = 4.35, *p* = .038, ηρ^2^ = .02. Among participants who were overweight and who read the embedded heteronomy-prime message, gain-framed message participants reported lower intentions than loss-framed message participants, *F*(1, 223) = 4.67, *p* = .032, ηρ^2^ = .02. In addition, among participants who were overweight and who read the gain-framed message, embedded autonomy-prime participants reported greater Time 1 intentions than embedded heteronomy-prime participants, *F*(1, 223) = 5.27, *p* = .023, ηρ^2^ = .02. Among participants who were overweight and who read the loss-framed message, embedded autonomy-prime participants reported marginally lower intentions than embedded heteronomy-prime participants, *F*(1, 223) = 3.77, *p* = .053, ηρ^2^ = .02. There were no other significant simple effects, all *p*s>.10. The interaction between Embedded Prime and Frame for participants who were overweight is shown in Figure 2.

For Time 2 intentions, there was no main effect of Embedded Prime, *F*(1, 177) = 0.32, *p* = .574, ηρ^2^<.01, but a significant effect of Frame *F*(1, 223) = 5.68, *p* = .018, ηρ^2^<.03, with participants who read the gain-framed message reporting greater Time 2 intentions (*M* = 4.72, *SE* = 0.15) than participants who read the loss-framed message (*M* = 4.22, *SE* = 0.14). There was no significant Embedded Prime×Frame interaction, *F*(1, 177) = 1.33, *p* = .250, ηρ^2^<.01. However, there was a significant Embedded Prime×Frame×BMI interaction, *F*(1, 177) = 5.93, *p* = .016, ηρ^2^ = .03. For participants with who were overweight, there was a significant Embedded Prime×Frame interaction, *F*(1, 107) = 6.01, *p* = .016, ηρ^2^ = .05, whereas there was no significant Prime×Frame interaction for participants who were normal weight, *F*(1, 73) = 0.47, *p* = .494, ηρ^2^<.01. Simple main effects analysis indicated that among participants who were overweight and who read the embedded autonomy-prime message, gain-framed message participants reported greater Time 2 intentions than did loss-framed message participants, *F*(1, 177) = 6.66, *p* = .011, ηρ^2^ = .04. In addition, among participants who were overweight and who read the loss-framed message, embedded autonomy-prime participants reported lower Time 2 intentions than embedded heteronomy-prime participants, *F*(1, 177) = 6.67, *p* = .011, ηρ^2^ = .04. There were no other significant simple effects, all *p*s>.10.

#### Predicting behavior

For Time 2 snacking behavior, there was no main effect of Embedded Prime, *F*(1, 170) = 0.02, *p* = .904, ηρ^2^<.01, or Frame, *F*(1, 170) = 0.32, *p* = .573, ηρ^2^<.01, and no significant Embedded Prime×Frame interaction, *F*(1, 170) = 0.46, *p* = .500, ηρ^2^<.01. However, there was a significant Embedded Prime×Frame×BMI interaction, *F*(1, 170) = 4.01, *p* = .047, ηρ^2^ = .02. There was no significant Prime×Frame interaction for either participants who were overweight, *F*(1, 104) = 2.62, *p* = .109, ηρ^2^ = .03, or normal weight, *F*(1, 69) = 0.27, *p* = .602, ηρ^2^<.01. However, simple main effects analysis indicated that among participants who were overweight and who read the embedded heteronomy-prime message, those who read the gain-framed message reported greater snacking than those who read the loss-framed message, *F*(1, 170) = 5.15, *p* = .025, ηρ^2^ = .03. In addition, among participants who were overweight and who read the gain-framed message, those who read the embedded autonomy-prime message reported marginally lower snacking than those who read the embedded heteronomy-prime message, *F*(1, 170) = 3.62, *p* = .059, ηρ^2^ = .02. There were no other significant simple effects, all *p*s>.01.

### Discussion

Study 2 examined whether autonomy and heteronomy primes that were embedded within the health information would influence the effect of gain-framed vs. loss-framed health messages about the negative health consequences of unhealthy high calorie snack consumption on participant’s intentions to avoid snacking, and their snacking behavior seven days later. The results showed that for those participants who were overweight or obese (with BMI over 25), the gain-framed message was more effective in promoting intentions to avoid high calorie snacks and the avoidance of high calorie snacks when embedded autonomy (compared to heteronomy) primes were present, and the loss-framed message was more effective when embedded heteronomy (compared to autonomy) primes were present. Unlike in Study 1, the effect of the frame condition for heteronomy primed participants occurred for both intentions and behavior. The results suggest that the extent to which autonomy or heteronomy is highlighted in the health message is an important predictor of whether a gain-framed or loss-framed is more effective in motivating intention and behavior change for participants who were overweight or obese.

Study 2 extended the findings of Study 1 by showing that non-conscious motivational primes can be incorporated within a health message to elicit an effect on intentions and behavior. However, the effect sizes of the prime in Study 2 were somewhat weaker than those in Study 1, particularly for our behavior measure. This is likely to be lower exposure of participants to the autonomy or heteronomy words, with fewer words related to autonomy and heteronomy incorporated in the message compared to in the priming task. Future research would benefit from using a greater number of primes embedded in the message, which may help to strengthen the effect of the embedded primes due to repeated exposure to the autonomy and heteronomy related words. Study 2 further extended the Study 1 findings by examining the moderating influence of BMI. In Study 2, the different effects of the health messages on intentions and behavior were only present for participants to whom the message was most relevant (i.e., for participants who were overweight or obese). For participants within the healthy BMI range, the embedded-prime and frame conditions did not influence subsequent intentions to avoid high calorie snacks or snacking behavior.

## General Discussion

Priming autonomy has been shown to increase autonomous self-regulation [31] [33] and therefore may reduce the perception of risk associated with failing to adhere to health recommendations. In addition, priming autonomy may reduce the perception that accepting the advice of others challenges self-integrity [33]. In accordance with this previous research, and as gain-framed messages are theorized to be more effective than loss-framed messages in promoting low risk behaviors [1] [2], priming autonomy in this study increased the effectiveness of gain-framed, but not loss-framed messages. In addition, priming heteronomy increased the effectiveness of the message in the loss-framed condition. This could be due to the heteronomy prime increasing the perceived risk of the behavior, as loss-framed messages have been suggested to be most effective when risk perceptions are high. These findings are consistent with recent research which found higher individual levels of autonomy to be associated with greater acceptance of gain-framed compared to loss-framed messages about fruit and vegetable consumption [29].

The results support previous research which has found no overall effects of message frame on persuasion [7] [8] [9] [10] and suggest that the weak, inconsistent effects of a gain-frame advantage when promoting low risk prevention behaviors found in meta analyses [6] [11] [12] may be due to a lack of consideration paid to the varying contexts which influence perceptions of risk. The application of prospect theory to health behavior change thus requires researchers to consider the factors found to moderate the effects of gain and loss-framed information on adherence to the recommended health behavior, such as the positive or negative mood of the recipient [16], emotional states of fear or anger [17], the self-efficacy of the reader [18], and the recipient’s motivational orientation [19]. In addition, the current findings suggest that the effectiveness of health messages may be increased if the autonomy supportive or coercive context is matched to the message frame. Although the current findings offer a useful insight into the context in which gain or loss-framed messages may be more effective for reducing high-calorie snacking behavior, there are some limitations to consider. Both Study 1 and Study 2 suffered a relatively high attrition rate (59% in Study 1, and 19% in Study 2. In addition, reporting of dietary intake is often problematic [39], and the single-item self-reported measure of snacking may have been unreliable. Additional measures such as a 24 hour food recall diary could have provided a more accurate account of participant’s snack intake [40]. The measure used in both Study 1 and Study 2 also focused on the number of high-calorie snacks consumed, and did not account for the nutritional value or portion size of each snack. Further research is also required to examine the potential mediating mechanisms underlying our findings. For example, an autonomy-prime coupled with a gain-framed message could increase positive affect or self-efficacy, which in turn may influence message acceptance. In addition, it would be beneficial to examine the role of autonomous motivation towards the health behavior. If the autonomy-prime, gain-framed message elicits greater autonomous motivation to conduct the behavior, this may elicit increased long term adherence to the behavior compared to the heteronomy-prime, loss-framed message, as research has shown that autonomous (vs. controlled) motivation is associated with greater persistence and commitment to a variety of health behavior [23] [24] [25] [26]. Although a heteronomy-prime, loss-frame message may motivate behavior change in the short term, this motivation may need to be internalized and integrated into the self in order to elicit longer term behavior change [20] [25]. In addition, it would be useful to determine whether the autonomy prime coupled with a loss-framed message elicits a reactance effect or result in more systematic or heuristic information processing due to the disparity between the prime and frame manipulations. Future research could also usefully explore the extent to which these findings are replicated for detection behaviors such as HIV testing and mammography and to examine whether the findings are similar for other protection behaviors such as fruit and vegetable consumption or exercise adherence. Given the success of the loss-frame coupled with the heteronomy prime in the current study, we would predict that a heteronomy prime may amplify the effects of loss-framed messages for detection behaviors.

The results have significant implications for the design of health promotion messages which persuade people to avoid eating high-calorie snacks. For example, autonomy and heteronomy related words could be introduced into health messages to ensure that the style of language used is correctly matched to the type of gain-framed vs. loss-framed information being conveyed. It is also possible that information which is perceived as from a source that is autonomy-supportive would be more effective if it were gain-framed, whereas information which is perceived as from a source that is coercive may be more effective if it were loss-framed [28]. Further research that examines these effects for other behaviors is needed to clarify whether these findings could extend to other prevention and detection behaviors. The findings suggest that further exploration of the role of autonomy in promoting health behaviors is warranted.

## Supporting Information

Figure S1 **Study 1 materials: Gain-framed vs. loss-framed health messages.**
(PDF)

Figure S2 **Study 2 materials: Examples of gain-framed messages with embedded autonomy or heteronomy primes.**
(PDF)
