# Supplementary material for: Promoting the Avoidance of High-Calorie Snacks: Priming Autonomy Moderates Message Framing Effects
Source: PLoS One. 2014 Jul 31;9(7):e103892. doi: 10.1371/journal.pone.0103892 (PMC4117640; doi:10.1371/journal.pone.0103892)
Supplement: Figure S1 — Study 1 materials: Gain-framed vs. loss-framed health messages. (PDF) [file pone.0103892.s001.pdf]

Figure S1. Study 1 materials: Gain-framed vs. loss-framed health messages.

Loss-framed health message	*Gain-framed health message*
Snack foods such as cakes, biscuits, chocolate, crisps, ice-cream and pastries are high in saturated fat and added sugar.<br>Evidence suggests that people who **eat lots of high calorie snacks** with high levels of saturated fat and added sugar, compared to those who avoid eating lots of snacks, are at **higher risk** of many serious **life-threatening diseases** and **lose** several potential health benefits.<br>For example, **research shows that people who EAT lots of snacks have a HIGHER RISK of:**<br>• **Heart Disease and** Stroke<br>• Type 2 Diabetes<br>• Cancers (e.g., bowel cancer)<br>**EATING lots of snacks can also mean you LOSE other HEALTH BENEFITS such as:**<br>• **Healthy looking skin and hair**<br>• **Healthy weight**<br>• **Increased energy and vitality**	Snack foods such as cakes, biscuits, chocolate, crisps, ice-cream and pastries are high in saturated fat and added sugar.<br>Evidence suggests that people who **avoid eating lots of high calorie snacks** with high levels of saturated fat and added sugar, compared to those who eat lots of snacks, are at **lower risk** of many serious life-threatening diseases and **gain** several potential health benefits.<br>For example, **research shows that people who AVOID EATING lots of snacks have a LOWER RISK of:**<br>• **Heart Disease and** Stroke<br>• Type 2 Diabetes<br>• Cancers (e.g., bowel cancer)<br>**AVOIDING EATING lots of snacks can also mean you GAIN other HEALTH BENEFITS, such as:**<br>• **Healthy looking skin and hair**<br>• **Healthy weight**<br>• **Increased energy and vitality**
