# Supplementary material for: Promoting the Avoidance of High-Calorie Snacks: Priming Autonomy Moderates Message Framing Effects
Source: PLoS One. 2014 Jul 31;9(7):e103892. doi: 10.1371/journal.pone.0103892 (PMC4117640; doi:10.1371/journal.pone.0103892)
Supplement: Figure S2 — Study 2 materials: Examples of gain-framed messages with embedded autonomy or heteronomy primes. (PDF) [file pone.0103892.s002.pdf]

**Figure S2: Study 2 materials: Examples of gain-framed messages with embedded autonomy or heteronomy primes.**

| *Gain-framed, embedded autonomy message* | *Gain framed, embedded heteronomy message* |
|---|---|
| **Choosing to avoid eating high calorie snacks**<br>Snack foods such as cakes, biscuits, chocolate, crisps, ice-cream and pastries are high in saturated fat and added sugar.<br>It is **up to you** whether you **choose** to avoid eating high calorie snacks. However, **evidence suggests that** people who **opt** to avoid eating high calorie snacks, compared to those that do not, are at lower risk of many serious life-threatening diseases.<br>For example, research shows that if you **decide** to **AVOID** eating high calorie snacks, you can **LOWER YOUR RISK** of:<br>• Heart Disease and Stroke<br>• High blood pressure<br>• High cholesterol<br>• Type 2 Diabetes<br>• Cancers (e.g., bowel cancer)<br>If you **elect** to **AVOID** eating high calorie snacks You can also **GAIN POTENTIAL HEALTH BENEFITS**, for example:<br>• Healthy looking skin and hair<br>• Healthy weight<br>• Increased energy and vitality<br>• Improved physical stamina<br>• Improved concentration on mental tasks<br>**The choice to avoid eating high calorie snacks is yours!** | **Requirement to avoid eating high calorie snacks**<br>Snack foods such as cakes, biscuits, chocolate, crisps, ice-cream and pastries are high in saturated fat and added sugar.<br>It is **essential** that you **should** avoid eating high calorie snacks. Furthermore, **evidence confirms** that people who avoid eating high calorie snacks, compared to those that do not, are at lower risk of many serious life-threatening diseases.<br>For example, research shows that if you **make yourself AVOID** eating high calorie snacks, you can **LOWER YOUR RISK** of:<br>• Heart Disease and Stroke<br>• High blood pressure<br>• High cholesterol<br>• Type 2 Diabetes<br>• Cancers (e.g., bowel cancer)<br>If you **force yourself** to **AVOID** eating high calorie snacks you can also **GAIN POTENTIAL HEALTH BENEFITS**, such as:<br>• Healthy looking skin and hair<br>• Healthy weight<br>• Increased energy and vitality<br>• Improved physical stamina<br>• Improved concentration on mental tasks<br>**Avoiding eating high calorie snacks is a must!** |
